# Early results of intra-articular micro-fragmented lipoaspirate treatment in patients with late stages knee osteoarthritis: a prospective study

**DOI:** 10.3325/cmj.2019.60.227

**Published:** 2019-06

**Authors:** Damir Hudetz, Igor Borić, Eduard Rod, Željko Jeleč, Barbara Kunovac, Ozren Polašek, Trpimir Vrdoljak, Mihovil Plečko, Andrea Skelin, Denis Polančec, Lucija Zenić, Dragan Primorac

**Affiliations:** 1 University Hospital Sveti Duh, Zagreb, Croatia; 2 Department of Radiology, St. Catherine Special Hospital, Zabok/Zagreb, Croatia; 3 Department of Orthopedics, St. Catherine Hospital, Zabok/Zagreb, Croatia; 4 Department of Public Health, School of Medicine, University of Split, Split, Croatia; 5 Specialty Hospital St. Catherine, Croatia; 6 Srebrnjak Children's Hospital, Zagreb, Croatia; 7 St. Catherine Specialty Hospital, Zabok/Zagreb, Croatia; 8 Eberly College of Science, The Pennsylvania State University, University Park, PA, USA; 9 University of Split, School of Medicine, Split, Croatia; 10 School of Medicine, Josip Juraj Strossmayer University of Osijek, Osijek, Croatia; 11 Faculty of Dental Medicine and Health Osijek, Josip Juraj Strossmayer University of Osijek, Osijek, Croatia; 12 Faculty of Medicine, University of Rijeka, Rijeka, Croatia; 13 Henry C. Lee College of Criminal Justice and Forensic Sciences, University of New Haven, West Haven, CT, USA

## Abstract

**Aim:**

To analyze clinical and functional effects of intra-articular injection of autologous micro-fragmented lipoaspirate (MLA) in patients with late stage knee osteoarthritis (KOA). Secondary aims included classifying cell types contributing to the treatment effect, performing detailed MRI-based classification of KOA, and elucidating the predictors for functional outcomes.

**Methods:**

This prospective, non-randomized study was conducted from June 2016 to February 2018 and enrolled 20 patients with late stage symptomatic KOA (Kellgren Lawrence grade III, n = 4; and IV, n = 16) who received an intra-articular injection of autologous MLA in the index knee joint. At baseline radiological KOA grade and MRI were assessed in order to classify the morphology of KOA changes. Stromal vascular fraction cells obtained from MLA samples were stained with antibodies specific for cell surface markers. Patients were evaluated at baseline and 12-months after treatment with visual analog scale (VAS), Western Ontario and McMaster Universities Osteoarthritis Index (WOMAC), and Knee Injury and Osteoarthritis Outcome Score (KOOS).

**Results:**

Three patients (15%) received a total knee replacement and were not followed up completely. Seventeen patients (85%) showed a substantial pattern of KOOS and WOMAC improvement, significant in all accounts. KOOS score improved from 46 to 176% when compared with baseline, WOMAC decreased from 40 to 45%, while VAS rating decreased from 54% to 82% (all *P* values were <0.001). MLA contained endothelial progenitor cells, pericytes, and supra-adventitial adipose stromal cells as most abundant cell phenotypes.

**Conclusion:**

This study is among the first to show a positive effect of MLA on patients with late stages KOA.

ISRCTN registration ID: ISRCTN13337022.

Knee osteoarthritis (KOA) is a disabling disease affecting around 250 million people worldwide. Risk factors that can facilitate KOA include age, genes, environment, and local trauma (1,2). Osteoarthritis involves deterioration of cartilage, subchondral bone, joint deformities, ligament and menisci damage, and surrounding muscle atrophy. Magnetic resonance imaging (MRI) enables detecting structural changes of KOA. It helps classify KOA according to the International Cartilage Repair Society scale in five stages, depending on the severity of the chondral lesion and surrounding subchondral bone. Runhaar et al (3) have demonstrated that MRI can be a valid tool for semiquantitative scoring of KOA. Magnetic Resonance Imaging Osteoarthritis Knee Score (MOAKS) can be used to quantify disease status. The gold standard in the diagnosis of KOA is the Kellgren-Lawrence classification. However, there are new paradigms in the KOA classification. Dell'Isola et al (4) presented a new KOA classification with regard to the phenotype. He allocated patients into groups characterized by specific disease mechanisms and analyzed their relation with demographic and health outcomes. In their study, 599 patients were selected and matched for six phenotypes: Minimal Joint Disease, Malaligned Biomechanical, Chronic Pain, Inflammatory, Metabolic Syndrome, and Bone and Cartilage Metabolism. The results showed that phenotype classification had an effect on WOMAC pain and physical function, which could mean that selecting patients by phenotype could give us better therapy outcomes (4). The complexity of KOA makes treatment difficult and partially unpredictable. Conservative approach of KOA treatment deploys non-steroidal anti-inflammatory drugs (NSAIDS), intra-articular steroid injections, hyaluronic acid, platelet rich plasma, and physical therapy. Surgical methods include joint preserving surgery for early KOA stages and knee replacement surgery for late stages (5-11). Current treatment strategies are a form of palliation and are aimed at providing symptomatic pain relief, failing to prevent the disease and/or improve tissue structure. However, recent reports on the outcomes of cell based therapies show that there is a potential to structurally remodel knee joint at a biochemical level, contributing to anabolic effects in tissues like bone and cartilage rather than to anti-inflammatory mechanisms (11-14). Adult mesenchymal stem cells (MSCs) have been in the last decade brought under the spotlight for various regenerative purposes. Their paracrine activity is thought to be one of the major mechanisms by which MSCs inhibit ischemia-caused apoptosis, scar formations, stimulate angiogenesis, and mediate anti-inflammatory and wound healing properties. Biological mediators secreted by MSCs have complex interaction when they regulate various regeneration processes. Pericytes, cells present on the micro vessels and capillaries in various tissues, are in *vivo* precursors of MSCs. Pericytes become activated during tissue injury and detach to become MSCs. Healing process is a site of busy cell action and interaction, initiated by MSCs signaling (15-18).

The application of MSCs in the treatment of early stages KOA can bring pain relief, improve knee joint function, and influence cartilage status while remaining safe (18-21). The aim of this study was to evaluate clinical and functional results of an intra-articular injection of autologous micro-fragmented lipoaspirate (MLA) in patients with severe KOA 12 months after the treatment. Secondary aims included classifying and determining cell types contained in the MLA, performing detailed MRI based classification of KOA, elucidating predictors for functional outcomes, and determining complication rates. We hypothesized that MLA treatment would improve clinical outcomes in patients with late stage KOA thus postponing TKR surgery.

## Patients and methods

This prospective, non-randomized, interventional, single-center, open-label clinical trial involved patients with primary late stage KOA who received a single intra-articular injection of autologous MLA. Inclusion and exclusion criteria and data collection procedure were the same as in our previous study (18). Briefly, the study enrolled patients with primary KOA from one center (to which all primary KOA patients were referred) who met the inclusion criteria (radiological Kellgren Lawrence grade III and IV; onset of symptoms of the index knee at six or more months ago; ability to follow the study instructions; age 40–85). The exclusion criteria were age <40 years or >85 years; chondromatosis or villonodular synovitis of the knee; recent trauma (<3 months) of the symptomatic knee; infectious joint disease; malignancy; pregnancy; anticoagulant therapy with prothrombin time (<0.70) or thrombocytopenia and/or coagulation disorder; and hypersensitivity to local anesthetics. Participants underwent detailed clinical history-taking, complete physical examination, and radiological assessment including plain x-rays (AP standing and LL knee projections), full-length weight bearing x-ray in the standing position in order to measure limb alignment, and MRI. Clinical and functional assessment included the visual analog scale (VAS), Western Ontario and McMaster Universities Osteoarthritis Index (WOMAC), and Knee Injury and Osteoarthritis Outcome Score (KOOS) pain scale at baseline and at 12-months follow-up. Twenty patients were enrolled and 20 knees were assessed. Eleven screened patients were not included because they did not meet enrollment criteria. No patients were excluded from the study. After having received detailed written and oral information about the study protocol, all patients gave informed consent. Each patient who entered the trial received a unique anonymous code. Data were collected in the data logbook. At baseline, we collected data on primary diagnoses and medical history, as well as patient demographic data (18). The recruitment period lasted from June 2016 to February 2017, with the follow-up period ending in February 2018. All procedures were standardized and implemented according to the standard operating procedure protocol. Primary outcome measure was avoiding TKR surgery. Secondary outcome measures were VAS, KOOS, and WOMAC results. The study protocol was approved by the local Institutional Review Board (IRB; authorization No: EP 001/2016). All the patients were enrolled after IRB approval had been obtained and some of them before finalization of the ISCRTN registration (February 24, 2017).

### KOA radiological classification

The Kellgren-Lawrence grading system classifies the severity of KOA on standard weight-bearing knee radiographs in two planes using five grades: grade 0 – no radiographic features of KOA are present; grade 1 – doubtful joint space narrowing (JSN) and possible osteophytic lipping; grade 2 – definite osteophytes and possible JSN on anteroposterior weight-bearing radiograph; grade 3 – multiple osteophytes, definite JSN, sclerosis, possible bony deformity; grade 4 – large osteophytes, marked JSN, severe sclerosis, and definitive bony deformity.

The severity of KOA in the study cohort was characterized on MRI by an experienced musculoskeletal radiologist (IB) using scoring system introduced by International Cartilage Research Society based on modified Outerbridge system divided in 5 stages according to cartilage lesions size and depth, as well as the appearance of the surrounding subchondral bone: grade 0 – normal cartilage; grade 1 – signal intensity alterations with an intact surface of the articular cartilage compared with the surrounding normal cartilage; grade 2 – partial thickness defect of the cartilage with fissures on the surface that do not reach subchondral bone or exceed 1.5 cm in diameter; grade 3 – fissuring of the cartilage to the level of the subchondral bone in an area with a diameter more than 1.5 cm; and grade 4 – exposed subchondral bone.

The MR images were performed on a 1.5T magnet (Avanto; Siemens, Erlangen, Germany) using a dedicated knee coil (Siemens, Erlangen, Germany). Proton density sequences with fat suppression [TR = 2700 ms; TE = 28 ms; Bandwidth = 326Hz; FOV = 170 mm; Matrix = 384 × 384; voxel size = 0.4 × 0.4 × 3mm; NEX = 2] in three planes were used to evaluate femoral, tibial, and patellar articular cartilage.

The status of cartilage was analyzed on seven different articular facets: medial and lateral femoral condyle, femoral trochlea, medial and lateral tibial condyle, and both patellar facets.

The presence of meniscal tears was analyzed in three different locations of each meniscus: anterior horn, corpus, and posterior horn. Meniscal tear was defined as linear signal intensity changes that reach meniscal surfaces at least 2 consecutive MR slices (slice thickness of 3 mm).

Associated subchondral bone marrow edema and joint effusion were analyzed as a reactive change with knee OA. Subchondral bone marrow edema was defined as an abnormal fluid signal seen within the bone marrow and joint effusion as a fluid signal intensity collection within the knee joint space on MRI.

### Transplantation and processing of micro-fragmented lipoaspirate

The patients were referred to the day surgery unit with an average admission of 3 h. The surgical part of the procedure was set in an operating theater and performed as described in our previous study (18). Briefly, patients were placed in a supine position; the abdominal skin was treated with Dermoguard® antiseptic lotion (Antiseptica, Pulheim, Germany), rinsed with Aqua pro injection solution (HZTM, Zagreb, Croatia), and dried out and disinfected with Skin-Des® solution (Antiseptica, Pulheim, Germany). The minimal invasive surgical procedure included an infiltration step, in which a total of 250 mL of saline solution prepared with a 40 mL of a 2% lidocaine solution (Lidokain®, Belupo, Koprivnica, Croatia) and 1 mL epinephrinhydrochloride (1mg/mL) (Suprarenin®, Sanofi-Aventis, Berlin, Germany) was injected in the abdominal subcutaneous adipose tissue. In the aspiration step, a standard lipoaspiration technique was performed, and the harvested fat was introduced into the Lipogems® ortho kit (Lipogems International SpA, Milan, Italy) according to the manufacturer’s instructions. The collected and processed final micro-fragmented adipose tissue product was transferred to 10 mL syringes and injected intra-articularly (5 mL) into the index knee.

### Stromal vascular fraction (SVF) isolation

Micro-fragmented lipoaspirate (MLA) specimens were stored overnight at room temperature (RT) protected from light. Samples were then digested with 0.1% collagenase type I in DMEM (both from Sigma-Aldrich, Saint Louis, MO, USA) in a shaking bath at 37°C for 45 minutes. After a 1:2 sample dilution with 2% fetal bovine serum (Biosera, Nuaille, France) in DMEM, the samples were filtered through a 100 μm cell strainer (BD Falcon, Corning, NY, USA) and centrifuged at 300 g for 10 minutes at RT. Supernatants were discarded and cell pellet was resuspended in 1 mL of the VersaLyse solution (Beckman Coulter, Miami, FL, USA). After 10 minutes, the samples were filtered through a 40 μm cell strainer (BD Falcon), centrifuged at 300 g for 10 minutes at RT, and the pellet was resuspended in DMEM. Cells were counted on the Sysmex XT1800 counter (Sysmex, Kobe, Japan).

### Flow cytometry

SVF cells obtained from MLA samples of KOA patients were stained with antibodies specific for the cell surface markers: CD31, CD34, CD45, CD73, CD90, CD105, CD146, labeled with PB, ECD, APC-AF750, PE, FITC, CD45-PC7, PC5.5 fluorochromes, respectively (kindly provided by Beckman Coulter). In parallel, dead cells were stained with Live/Dead Yellow Fixable Stain (ThermoFisher, Waltham, MA, USA). After 20 minutes, samples were fixed with 2% paraformaldehyde (Electron Microscopy Sciences, Hatfield, PA, USA) in PBS (Sigma-Aldrich), washed, and permeabilized with PermWash (BD Biosciences, San Jose, CA, USA). The cell nuclei were stained with the DRAQ7 dye (BioStatus, Shepshed, UK). The FlowLogic software (Inivai Technologies, Mentone, Australia) was used for the analysis of the FCS data files.

### Statistical analysis

Descriptive and analytic methods were used. The normality of distribution was tested by the Shapiro-Wilks test. Data are presented as mean and standard deviation. Paired data were analyzed using paired *t* test. We also used a forward model of linear regression, where all available clinical predictors were entered, in an attempt to identify the ones that were the most predictive for the outcome variable, in this case the change of the KOOS and WOMAC scores. The data were analyzed in R (*www.r-project.org*), with the level of significance set at *P* < 0.05.

## RESULTS

### Patients’ characteristics

The study enrolled 20 patients who met the inclusion criteria (15 men). Body mass index values were <30 in 13 patients, 30-35 in 5 patients, and >35 in 2 patients. In 19 patients, previous treatments (physical therapy, oral NSAIDs, or intra-articular injection of hyaluronic acid or steroids) had failed to relieve symptoms.

The pattern, distribution, and severity of cartilage tissue deterioration, bone marrow edema, effusion, and alignment as assessed by x-rays and MRI varied substantially at the baseline. Out of 20 knees, in 15 we observed varus deformities (>5° malalignment), 1 valgus knee with (>5° malalignment), and 5 knees with good alignment.

### Safety of intra-articular injection of autologous micro-fragmented lipoaspirate

No adverse events (AE) were observed in association with lipoaspiration or intra-articular injection. No infectious AEs related to intra-articular injection occurred during the follow-up.

### Clinical outcome

Out of 20 patients, 3 underwent TKA before the end of one-year period due to persistent high level of symptoms. The remaining 17 patients (85%) were analyzed in detail. The overall results showed a substantial and universal pattern of KOOS and WOMAC improvement, which was significant in all accounts (Table 1). In general, KOOS score improved by at least half, while WOMAC yielded a similar magnitude of decrease (Table 1).

**Table 1:** The initial comparison of all Knee Injury and Osteoarthritis Outcome Score (KOOS) and Western Ontario and McMaster Universities Osteoarthritis Index (WOMAC) related variables between baseline and 12-months follow up (mean ± standard deviation)

Clinical score	Baseline	12-months follow up	*P* (paired)	Average percent change
KOOS Pain_1	38.69 ± 17.34	64.57 ± 15.38	<0.001	+66.9
KOOS Symptom_1	47.76 ± 17.88	69.84 ± 15.91	<0.001	+46.2
KOOS ADL_1	39.6 ± 19.5	64.25 ± 17.84	<0.001	+62.2
KOOS Sport/Rec_1	16.25 ± 15.55	34.69 ± 20.85	0.003	+113.5
KOOS QOL_1	13.28 ± 12.68	36.7 ± 19.24	<0.001	+176.4
WOMAC PAIN_1	11.88 ± 3.76	6.5 ± 3.35	<0.001	-45.3
WOMAC STIFFNESS_1	4.31 ± 1.89	2.56 ± 1.46	0.001	-40.6
WOMAC PHYSICAL FUNCTION_1	39.19 ± 14.2	23.19 ± 10.85	<0.001	-40.8
WOMAC TOTAL SCORE_1	55.38 ± 18.83	32.25 ± 14.62	<0.001	-41.8
VAS resting_1	4.06 ± 2.35	0.75 ± 1.65	<0.001	-81.5
VAS movement_1	7.38 ± 1.41	3.38 ± 1.89	<0.001	-54.2

The use of the forward regression model for the KOOS and WOMAC, with a number of clinically relevant predictors resulted in a total of five significant models, four for KOOS pain and one for WOMAC pain (Table 2). Notably, these two models overlapped in the bone marrow edema of the anterior part of the knee joint between trochlea and patella (Table 2).

**Table 2:** Forward regression models predicting the extent of change of Knee Injury and Osteoarthritis Outcome Score (KOOS) and Western Ontario and McMaster Universities Osteoarthritis Index (WOMAC) scores for pain

KOOS pain	Predictor	Beta	*P*
Model 1	meniscus injury, lateral meniscus, corpus	-0.578	0.038
Model 2	meniscus injury, lateral meniscus, corpus	-0.780	<0.001
	meniscus injury, medial meniscus, horn	-0.709	0.001
Model 3	meniscus injury, lateral meniscus, corpus	-0.844	<0.001
	meniscus injury, medial meniscus, horn	-0.792	<0.001
	regular use of NSAID*	-0.332	0.014
Model 4	meniscus injury, lateral meniscus, corpus	-0.746	<0.001
	meniscus injury, medial meniscus, horn	-0.855	<0.001
	regular use of NSAID	-0.369	0.001
	bone marrow edema, patellofemoral joint	-0.295	0.003
WOMAC pain	bone marrow edema, patellofemoral joint	-0.668	0.013

*NSAID – nonsteroidal anti-inflammatory drugs.

### Immunophenotyping analysis of stromal vascular fraction from micro-fragmented lipoaspirate samples by polychromatic flow cytometry

**Figure 1:**
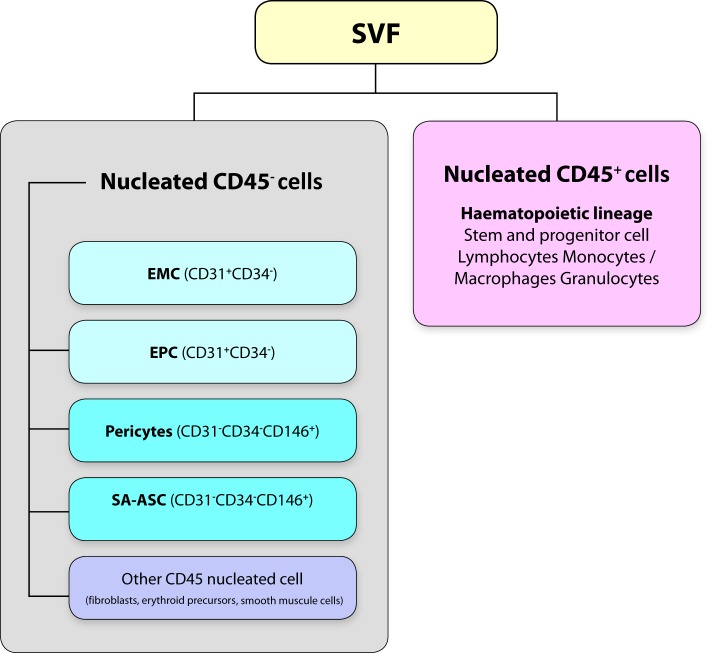
Summarized results of main imunophenotypes in the stromal vascular fraction (SVF) nucleated cell populations from microfragmented adipose tissue isolated from 20 osteoarthritic patients. EMC – endothelial mature cells, EPC – endothelial progenitor cells, SA-ASC – supra-adventitial adipose stromal cells.

The viability of nucleated CD45^-^ and CD45^+^ cells in SVF from MLA was 95% ± 3.6. Four main CD45^-^ subpopulations were CD31^-^CD34^-^CD146^+^ pericytes, CD31^-^CD34^+^CD146^-^ supra-adventitial adipose stromal cells (SA-ASC), CD31^+^CD34^+^ endothelial progenitor cells (EPC), and CD31^+^CD34^-^ mature endothelial cells (EM) (Figure 1). The determined subpopulations showed variable levels of the mesenchymal markers CD73, CD90, and CD105 (Polancec et al, unpublished data).

## DISCUSSION

In this study, we reported the results for 20 patients with late stages KOA receiving intra-articular micro-fragmented autologous fat tissue transplantation. Patients included in the study had grade III and IV KOA with high level of symptoms and pain at the baseline. Before study enrollment, all the patients had been candidates for a TKR surgery. Our study showed that MLA treatment relieved pain and clinical symptoms in 85% of patients with late stages KOA for one year. We should emphasize that there are only a few studies that showed a positive effect of adipose tissue-originated mesenchymal stem cells (Ad-MSCs) on patients with high grade KOA. The results of our study are in line with the results of some previous studies. Koh et al (22) found that Ad-MSCs exhibited effects on cartilage healing, reduced pain, and improved function in KOA patients evaluated by second look arthroscopy and clinical outcome scores. Jo et al (23) concluded that intra-articular injections of Ad-MSCs in KOA improved function and reduced cartilage defects. The study of Yokota et al (24) involved 13 patients (11 OA grade IV and 2 OA grade II according Kellgren-Lawrence scale). Based on the VAS, Japanese Knee Osteoarthritis Measure, and WOMAC score they found a significant improvement in the first month after the procedure and a small numerical improvement after six months. The results obtained in our study provide a good basis for prospective randomized controlled clinical trials with the aim to evaluate the use of autologous MLA containing AD-MSCs in the treatment of higher-grade KOA. Before adipose tissue microfragmentation was established, mechanical procedures had been considered as low-yield methods for obtaining SVF compared with enzymatic methods (25). However, MLA is rich in SVF cells with high regenerative capacity, as confirmed in our study.

**Figure 2:**
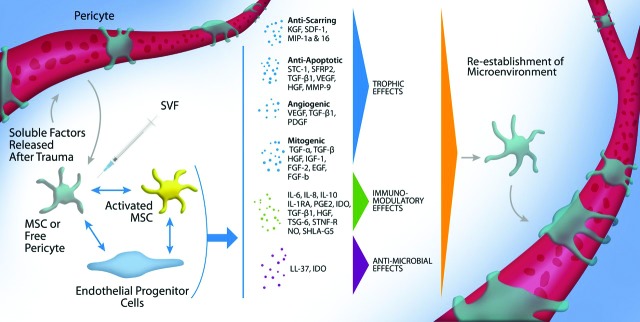
Pericytes are stimulated by soluble growth factors and chemokines to become activated mesenchymal stem cells (MSCs), and probably in interaction with endothelial progenitors both cell types respond to the microenvironment by secreting trophic (mitogenic, angiogenic, anti-apoptotic, or scar reduction), immunomodulatory or antimicrobial factors. After the microenvironment is re-established, MSCs return to their native pericyte state attached to blood vessels. SVF – stromal vascular fraction. Murphy BM, Moncivais K and Caplan IA. Mesenchymal stem cells: environmentally responsive therapeutics for regenerative medicine. Experimental and Molecular Medicine (2013) 45, e54; doi:10.1038/emm.2013.94 (Used and adapted with permission of Prof. Arnold I Caplan and publisher’s permission).

Our study suggests that application of autologuos micro-fragmented adipose tissue with SVF in patients with KOA increases GAG levels in hyaline cartilage, consequently reducing pain and improving movement abilities (18). SVF from adipose tissue contains SA-ASC, pericytes, EPC, EMC, and leukocytes. There are at least two possible mechanisms through which MSCs exert their effect and that might explain the success of SVF-treatment of KOA. The first one assumes that MSCs differentiate into chondrocytes, while the second one suggests that MSCs stimulate chondrocytes by paracrine secretion of bioactive factors. As our results revealed that SVF contained a respectable ratio of EPC (Polancec et al., unpublished data), this sheds a new light on the MSC-mediated cartilage tissue repair and regeneration that assumes cooperation with EPC. EPC represent important players implicated in vascularization as a prerequisite for wound repair and tissue regeneration (26,27). Several *in vitro* and *in vivo* studies have demonstrated proliferative, proangiogenic, and vasculogenic effects of MSC-EPC interactions (28-31), as well as differentiation commitment (32,33). A crosstalk between MSC-EPC, which can involve both paracrine mechanism and/or cell-cell contact (*via* PDGF-PDGFR and Notch-signaling) (29,34), seems to occur in different conditions of tissue inflammation (35), suggesting their interplay and beneficial effects also in cartilage tissue repair and regeneration. The fact that in our study EPCs outnumber MSCs indicates their inevitable involvement in the observed effect of the SVF-mediated cartilage treatment. Therefore, we hypothesize that a plethora of cytokines, chemokines, and growth factors, ie, bioactive molecules hitherto ascribed to the activated MSC, acting as medicinal signaling cells (36), perhaps stem from a synergistic effect of pericyte-MSC-EPC interactions (Figure 2).

Osteoarthritis primarily affects cartilage tissue, which is best diagnosed and described by MRI. However, radiography with standard x-rays is limited when it comes to recognizing early degenerative changes. The delayed gadolinium-enhanced MRI of cartilage technique assessing proteoglycan content has shown potential in evaluating the macromolecular status of normal, degenerated, and regenerated articular cartilage (18). Runhaar et al showed that MRI imaging can be a valid tool for semiquantitative scoring of KOA. MOAKS is primarily applicable to quantify disease status, which could lead to better treatment outcomes (3). Five Kellgren-Lawrence stages based on AP weight bearing x-rays merely describe pathological changes that a joint undergoes over time but do not provide a sufficient insight into treatment allocation. Dell’Isola et al (4) suggested a new classification based on phenotypes. All patients (599) were classified into predefined groups characterized by specific variables and assigned phenotype. At baseline and after 24 months WOMAC score was assessed. Both, WOMAC pain and physical function have been influenced by KOA phenotype. Assigning a phenotype to patients could improve clinical decision making and optimal treatment strategies (4). In our study, we used MRI to describe KOA morphology by assessing cartilage damage, bone marrow edema appearance and distribution, joint effusion, and location of the meniscus rupture. In our attempt to define KOA phenotype more accurately, we showed an association between baseline parameters and treatment results. Bone marrow edema noted on the knee MRI scans at baseline was shown to be a good predictor at both KOOS and WOMAC subscales for pain in terms of improvement after one year. In our previous study, application of MLA increased GAG content (18,37,38), implicating an effect on patellofemoral joint that bears high shear force loads. It has been shown that lipoaspirate with MSCs along with platelet-rich plasma has a positive effect in an early stage patellofemoral joint OA (39). Paracrine effect of cells from micro-fragmented fat tissue on chondrocytes to produce extracellular matrix might influence the quality of subchondral bone in patellofemoral joint, thus relieving pain captured by WOMAC and KOOS pain subscales. Recent results have shown that MLA has a long-lasting anti-inflammatory property, most likely influenced by a combination of cytokines secreted by SVF cells, primarily by MSCs (40). MLA is not a mechanically obtained SVF, it can serve as a natural scaffold for drug delivery (41). Even though we presented significant results, it has to be acknowledged that our study has certain limitations particularly due to a lack of a control group.

In conclusion, the present prospective study suggests that intra-articular injection of micro-fragmented fat tissue decreases clinical symptoms in patients with late stage KOA, without observed AE. Additional randomized clinical studies are warranted to improve clinical practice especially related to different KOA phenotypes.
